# Gut Microbiome in the Progression of NAFLD, NASH and Cirrhosis, and Its Connection with Biotics: A Bibliometric Study Using Dimensions Scientific Research Database

**DOI:** 10.3390/biology12050662

**Published:** 2023-04-27

**Authors:** Salvatore Pezzino, Maria Sofia, Chiara Mazzone, Sergio Castorina, Stefano Puleo, Martina Barchitta, Antonella Agodi, Luisa Gallo, Gaetano La Greca, Saverio Latteri

**Affiliations:** Department of Surgical Sciences and Advanced Technologies “G. F. Ingrassia”, Cannizzaro Hospital, University of Catania, 95123 Catania, Italy

**Keywords:** gut microbiome, NAFLD, NASH, cirrhosis, bibliometric analysis, biotics, prebiotic, probiotic, symbiotic, Dimensions database, VOSviewer

## Abstract

**Simple Summary:**

There is accumulating evidence that gut microbiome dysbiosis is associated with the etiopathogenesis of nonalcoholic fatty liver disease (NAFLD), from the onset of the disease to the progressive stages of nonalcoholic steatohepatitis (NASH) and cirrhosis. Furthermore, probiotics, prebiotics, and synbiotics have shown promise in restoring dysbiosis and lowering clinical indicators of disease in a number of trials, both preclinical and clinical. Additionally, postbiotics and parabiotics have recently garnered some attention. The purpose of this bibliometric analysis was to assess, using the Dimensions database, recent publishing trends concerning the role of the gut microbiome, in the progression of NAFLD into NASH and cirrhosis, and its connection with biotics (prebiotics, probiotics, symbiotics, postbiotics, and parabiotics).

**Abstract:**

There is growing evidence that gut microbiota dysbiosis is linked to the etiopathogenesis of nonalcoholic fatty liver disease (NAFLD), from the initial stage of disease until the progressive stage of nonalcoholic steatohepatitis (NASH) and the final stage of cirrhosis. Conversely, probiotics, prebiotics, and synbiotics have shown promise in restoring dysbiosis and lowering clinical indicators of disease in a number of both preclinical and clinical studies. Additionally, postbiotics and parabiotics have recently garnered some attention. The purpose of this bibliometric analysis is to assess recent publishing trends concerning the role of the gut microbiome in the progression of NAFLD, NASH and cirrhosis and its connection with biotics. The free access version of the Dimensions scientific research database was used to find publications in this field from 2002 to 2022. VOSviewer and Dimensions’ integrated tools were used to analyze current research trends. Research into the following topics is expected to emerge in this field: (1) evaluation of risk factors which are correlated with the progression of NAFLD, such as obesity and metabolic syndrome; (2) pathogenic mechanisms, such as liver inflammation through toll-like receptors activation, or alteration of short-chain fatty acids metabolisms, which contribute to NAFLD development and its progression in more severe forms, such as cirrhosis; (3) therapy for cirrhosis through dysbiosis reduction, and research on hepatic encephalopathy a common consequence of cirrhosis; (4) evaluation of diversity, and composition of gut microbiome under NAFLD, and as it varies under NASH and cirrhosis by rRNA gene sequencing, a tool which can also be used for the development of new probiotics and explore into the impact of biotics on the gut microbiome; (5) treatments to reduce dysbiosis with new probiotics, such as *Akkermansia*, or with fecal microbiome transplantation.

## 1. Introduction

Between 500 and 1000 different species of commensal bacteria, most of which are anerobic, reside in the human intestinal tract, forming what is known as the “gut microbiome” [1,2]. Dysbiosis of the gut microbiome has been linked to a variety of diseases, including chronic inflammation [3], neurodegenerative and ocular diseases [4,5], and chronic liver diseases [6,7,8]. Research has linked dysbiosis in the gut microbiome to the etiology of nonalcoholic fatty liver disease (NAFLD), a disorder that is produced by the deposition of fat within the liver cells even in the absence of alcohol usage, and its progression to nonalcoholic steatohepatitis (NASH), which is defined histologically by hepatocyte injury, inflammation, and varying degrees of fibrosis, until the last stage of cirrhosis [6,7,8,9]. In addition, there is emerging evidence from both preclinical and clinical research showing the disruption of the gut–liver axis (the communication and feedback loop between the gastrointestinal and hepatic systems) which plays a major role in NAFLD pathogenesis and its progression to NASH and cirrhosis, and that gut dysbiosis is a key factor in the development of this dysfunction [10,11,12,13]. Alteration of the gut–liver axis is characterized by a number of pathogenic mechanisms, including the weakening of the gut barrier and the rise in intestinal permeability, which result in endotoxemia and inflammation, as well as alterations in bile acid profile and metabolite levels produced by the gut microbiome [10,12,13]. Biotics, such as probiotics, prebiotics, and synbiotics, on the other hand, have shown promise in restoring dysbiosis [14,15,16]. Probiotics are defined as “live bacteria that, when administered in suitable proportions, provide health benefits to the host” [17]. Prebiotics are a type of substrate that is beneficial to human health because they are used selectively by host bacteria [17,18]. Synbiotics are formulated mixes of prebiotics and probiotics that work together to maximize their health benefits [14]. Several preclinical and clinical studies have indicated a positive effect of probiotics, prebiotics, and synbiotics in reversing gut dysbiosis and in clinical markers of the NAFLD disease and derived pathological spectrum [15,19]. In animal models, probiotics effectively decreased NAFLD dysbiosis, fatty acid synthesis, inflammation, and metabolic endotoxemia [20,21]. Insulin, glucose, triglyceride, and cholesterol levels, as well as transaminase activity, have all been measured and found to be affected by prebiotics, proving their impacts on carbohydrate and lipid metabolisms [22,23]. In mouse and human investigations, prebiotics, such as oligofructose, fructo-oligosaccharides, and isomalto-oligosaccharides, lowered LPS and cytokine levels, improved insulin resistance, decreased inflammatory and hepatic indicators, and decreased steatohepatitis [24,25]. Multiple human studies utilizing synbiotics in patients with NAFLD or NASH have demonstrated considerable improvements in hepatic steatosis and fibrosis compared to placebo, but changes in blood indicators of inflammation or liver injury remain heterogeneous [23,26,27]. Therefore, the use of these bioactive compounds for the prevention of NAFLD, NASH, and cirrhosis or as a complementary strategy in its treatment appears to be of significant interest, despite the lack of an approved pharmacological treatment for NAFLD/NASH. Other biotics, such as postbiotics and parabiotics, have recently garnered some attention [28]. Postbiotics are the cell-free supernatants of probiotics, which contain a wide variety of metabolic products, including enzymes, secreted proteins, short-chain fatty acids, vitamins, secreted biosurfactants, amino acids, peptides, and organic acids. Paraprobiotics, on the other hand, are the inactive microbial cells of probiotics [28,29]. Although it has been established the beneficial effects of probiotics, their broad application in the food and pharmaceutical industries has been hampered by technological obstacles, such as viability controls. Therefore, paraprobiotics and/or postbiotics, which are macromolecules derived from probiotics but are not themselves living organisms, are therefore gradually replacing viable probiotic bacteria as the focal point of attention [29]. There are now several pieces of evidence of the usefulness of paraprobiotic and postbiotics in several diseases, including NAFLD and in delaying its progression to cirrhosis [30,31].

The term “bibliometric analysis” is used to describe a comprehensive and up-to-date analysis of published works by examining variables, such as publication volume, authorship, geographical focus, citation, and keyword density, over a certain time frame. It gives researchers a detailed overview and updates on the field’s current state [32,33]. Recent bibliometric studies using the Web of Sciences database [34] have examined emerging trends and hotspots in the area of metabolic-dysfunction-associated fatty liver disease (MALD) [35] and the field of gut microbiota and MALD [36]. To further explore the role of the gut–liver axis in NAFLD, Yang et al. [37] analyzed global publication patterns and research hotspots. Different from the aforementioned studies, the purpose of our bibliometric analysis is to specifically evaluate recent publishing trends from 2002 to 2022 concerning the relationships between the gut microbiome, all stages of liver disease, from NAFLD to cirrhosis, and their connections with biotics (prebiotics, probiotics, symbiotics, postbiotics, and parabiotics) using the “Dimensions” database [38,39,40] in order to provide a comprehensive overview of the field of study, keep academics up to date on the most recent research trends, and offer crucial information that will be useful for exploring new research directions.

## 2. Materials and Methods

We performed a comprehensive literature search using the free access version Dimensions database [38,39,40]. Dimensions is a global academic database with about 1.4 billion citations, datasets, patents, and policy papers from several million scholarly publications [38]. We used the appropriate keywords to identify relevant literature from 2002 to 2022 on the connections between the gut microbiome, all phases of liver disease ranging from NAFLD to cirrhosis, and biotics (prebiotics, probiotics, symbiotics, postbiotics, and parabiotics). To prevent bias caused by continuous database modifications, the extraction and export of documents should be completed within one day. The date of the retrieval was 25 January 2023. The research string used was the following: (((microbiome) OR (microbiota) OR (dysbiosis) OR (flora) OR (microflora) OR (bacteria) OR (microorganism) OR (microbe)) AND ((gut) OR (intestine) OR (intestinal) OR (gastrointestine) OR (gastrointestinal) OR (“gut liver axis”) OR (“gut-liver axis”) OR (gastro-intestine) OR (gastro-intestinal))) AND ((NAFLD) OR (“non-alcoholic fatty liver disease”) OR (“nonalcoholic fatty liver disease”) OR (“non alcoholic fatty liver disease”) OR (NASH) OR (“non-alcoholic steatohepatitis”) OR (“nonalcoholic steatohepatitis”) OR (“non alcoholic steatohepatitis”) OR (cirrhosis)) AND ((probiotic) OR (biotic) OR (prebiotic) OR (synbiotic) OR (paraprobiotic) OR (postbiotic) OR (Lactobacillus) OR (Lactobacilli) OR (Bifidobacterium) OR (Streptococcus) OR (Streptococci) OR (Lactococcus) OR (Lactococci) OR (Bifidobacteria) OR (Akkermansia) OR (Saccharomyces) OR (Enterococcus) OR (Enterococci) OR (Bacillus) OR (Bacilli) OR (Ruminococcus) OR (Ruminococci)). Data mining was performed in the title and abstract and the publication type was “Article”.

By thoroughly examining the retrieved publications, we verified the efficacy of our search strategy. The information extracted from the Dimensions database was collected and stored in CSV format. The free access version of Dimensions provides several features that can be used in conjunction with the software VOSviewer (version 1.6.18) [41] to perform the processing and map visualization of the following datasets (in order in the article): publication trend, country productivity and cooperation, institution productivity and cooperation, author productivity and cooperation, co-authorship analysis, citations analysis, analysis of most cited journal and article, analysis of document co-citation reference, and the co-occurrence map based on text data in the title and abstract fields.

## 3. Results

### 3.1. Publication Trends and Country Productivity/Cooperation

**Figure 1:**
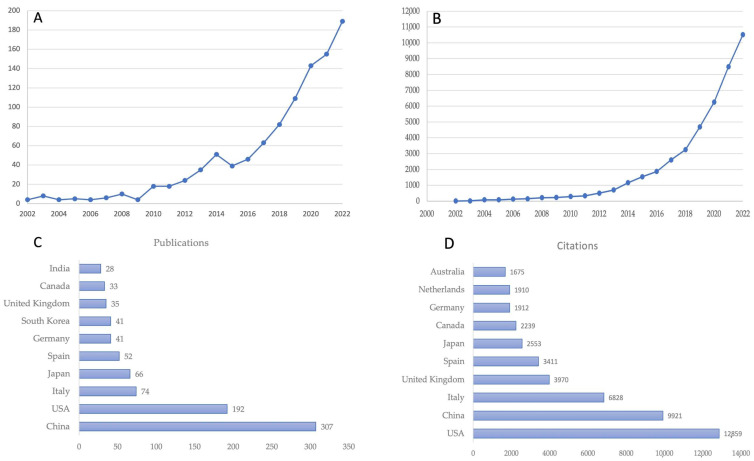
The number of publications (**A**) and citations (**B**) between 2002 and 2022; the top 10 most productive countries in terms of publications (**C**) and citations (**D**), in ascending order. All numbers are derived from Dimensions on 25 January 2023.

Based on the parameters of the search, 1015 articles covering the years 2002–2022 were retrieved from the Dimensions database. Over the past decade, especially the last five years, the number of publications in this sector has increased rapidly (Figure 1A), demonstrating that the intricate linkage encompassed in the gut microbiome, all stages of liver disease from NAFLD to cirrhosis, and biotics gained worldwide attention. Figure 1A displays the publication trend through time, which may be divided into three “ages”: the period from 2002 to 2009, during which only 41 pieces of literature were published, the period from 2010 to 2016, during which 231 publications were made, and the thriving period beginning in 2017, during which 741 publications (72.8%) were made. The number of citations in this field has been soaring, since 2018 going from about 3000 citations up to about 11,000 in 2022 (Figure 1B). Between 2002 and 2022, publications relating to the specified field of research were disseminated in 59 different nations. Panels C and D of Figure 1 display respectively the top 10 countries in terms of the total number of publications and the total number of citations.

**Figure 2:**
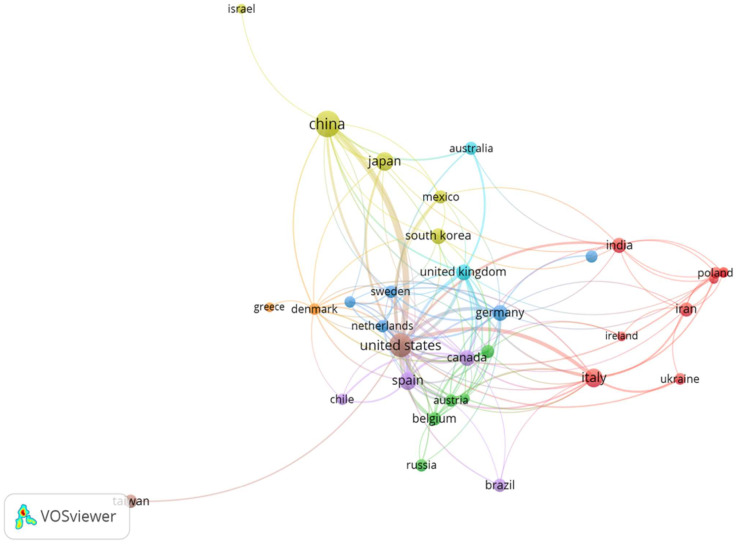
Cooperation network among the leading nations based on co-authorship productivity over the past two decades. The diverse colors symbolize the different clusters established by the groups of countries; there are eight separate clusters of cooperative relationships between nations, while the size of the circle is based on the countries’ productivity in the field; the wider the circle of each country, the greater the number of articles bearing its authorship. Objects’ closeness also reflects the degree of their connectedness.

The software VOSviewer was used to investigate cooperation across countries. Figure 2 depicts the 32 countries with the highest co-authorship productivity. The minimum number of documents and citations is set to four. The co-authorship analysis of countries reveals the collaboration between countries in this field as well as the level of collaboration. The larger circles reflect the most productive countries in the field; the thickness and length of the links between nodes represent the cooperative relationship between countries. The proximity of the objects also indicates the degree of their connection. Colors represent the clustering of the items. China, the USA, and Italy dominate the scientific scenario in this field both in terms of the number of publications and in terms of the number of citations. There are eight distinct clusters of cooperative relationships between nations, each distinguished by a distinct color. The United States had the highest overall link strength with 192 documents and a link strength of 125, followed by China with 307 documents and a total link strength of 57. Spain came in third place with 52 documents and 50 total link strengths. The United Kingdom, with 35 documents and a link strength of 43, is second, followed by Canada, with 33 documents and a link strength of 40, and Italy, with 74 documents and a link strength of 39.

### 3.2. Institution’s Productivity and Cooperation

**Figure 3:**
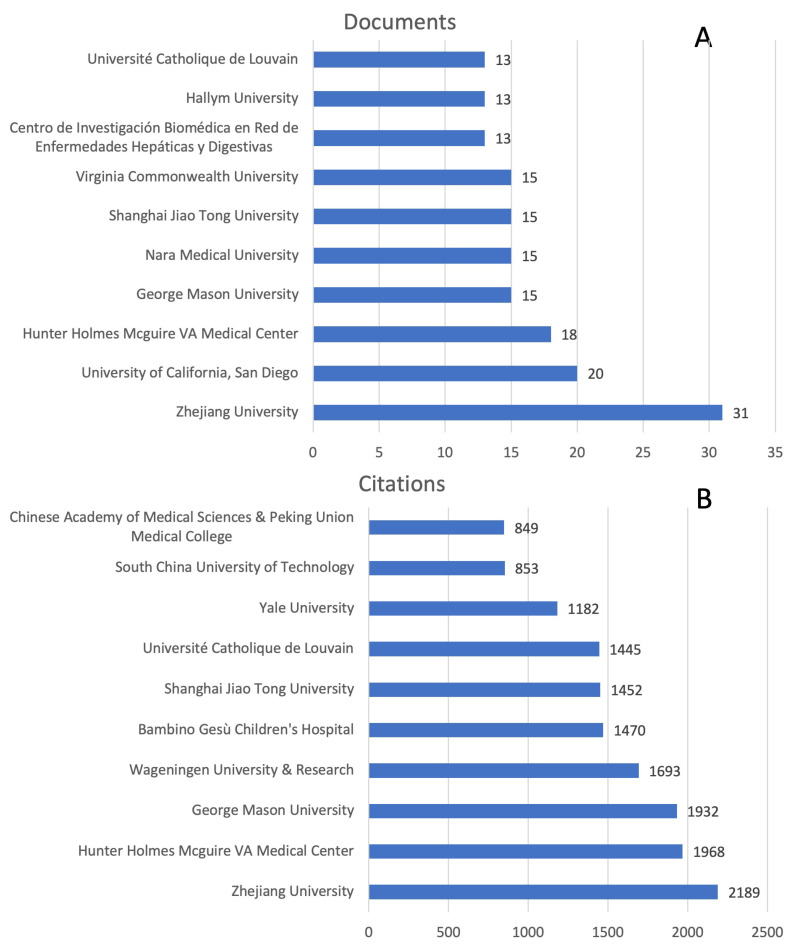
List of the most productive institutions in terms of publications (**A**) and citations (**B**), in ascending order. All numbers are derived from Dimensions on 25 January 2023.

The research on the role of the gut microbiome in the progression of NAFLD, NASH and cirrhosis, and its connection with biotics was supported by 1118 institutions. The top 10 institutions with the highest number of publications and citations are listed in Figure 3. The most prolific organization for publication is Zhejiang University (China), with 31 documents. The second and third positions are occupied by the University of California (San Diego, CA, USA) and Hunter Holmes Mcguire VA Medical Center (USA), with 20 and 18 publications, respectively. In the fourth position, we found several institutions with 15 publications: George Mason University, Nara Medical University, Shanghai Jiao Tong University, and Virginia Commonwealth University. The most cited institutions are, in order, Zhejiang University with 2189 citations, Hunter Holmes Mcguire VA Medical Center with 1968 citations, Wageningen University & Research (The Netherlands) with 1932 citations, and Wageningen University & Research with 1693 citations.

**Figure 4:**
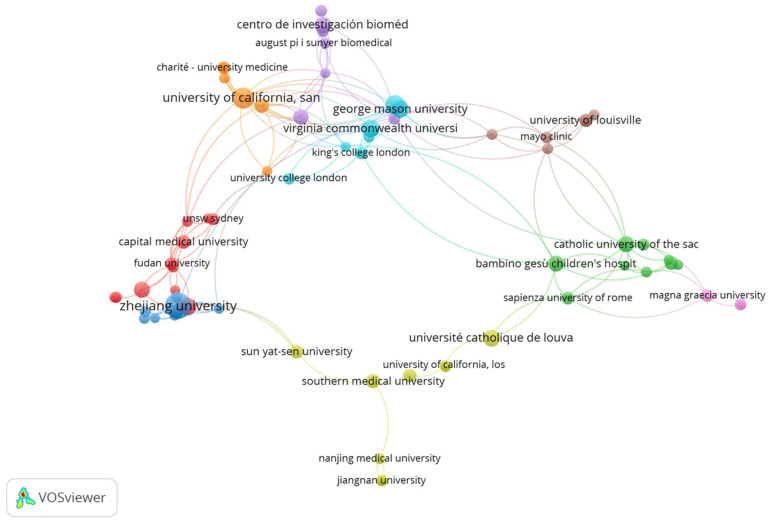
Collaboration network based on co-authorship productivity over the last 20 years between the major institutions. The different colors represent the different clusters formed by the groups of institutions; the degree of connection between the objects is also indicated by their proximity. The map depicts a network of nine clusters distinguished by different colors, which aid in understanding how the various institutions collaborate.

The software VOSviewer was used to examine and map the cooperation between institutions. Figure 4 depicts the larger aggregate of 69 institutions with active cooperation. Each concentric circle on the map symbolizes an organization, and the thickness of the linkages denotes the level of interaction between institutions. The size of the concentric circle indicates the number of publications produced by each organization, while the presence of link lines signifies active collaboration. The density of the connecting lines represents the level of cooperation between institutions. The map reveals a network of nine clusters distinguishable by different colors that help us understand how the various institutions cooperate.

### 3.3. Authors’ Productivity and Cooperation

Approximately 5663 authors contributed to the publication outputs between 2002 and 2022 (Table 1). The first prolific author is Bajaj, J. from Virginia Commonwealth University (USA) with 25 publications; the second position is occupied by Gillevet, P.M. and Sikaroodi, M., both from George Mason University (USA) with 15 publications. Su, K.T. from Hallym University (Republic of Korea) and Li, L. from Zhejiang University (China) are both in third place with 12 publications. The most cited authors of the top 20 are again Bajaj, J. with 2375 citations, Gillevet, P.M. and Sikaroodi, M. both with 1932 citations, and Hylemon, P.B. from Virginia Commonwealth University (USA) with 1347 citations.

**Figure 5:**
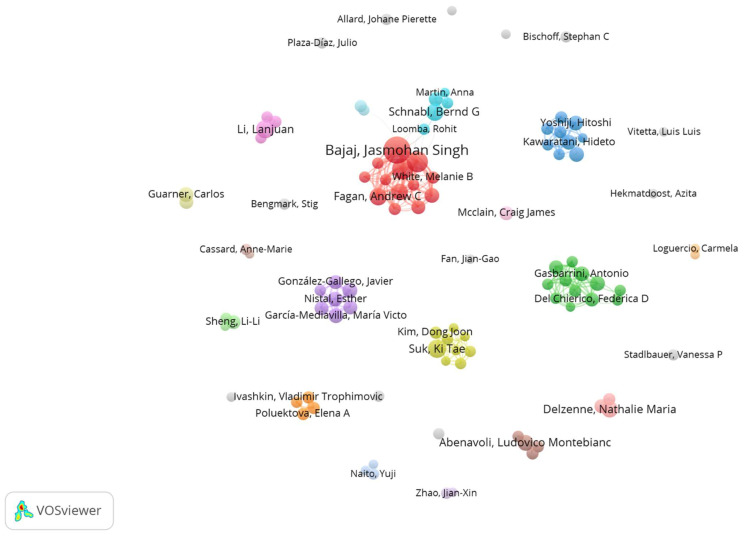
Analysis of the co-authorship of the top 100 authors. The relationship between scientists is defined by the number of articles they have co-authored, which provides clear information about existing collaborations. The diameter of the concentric circle represents the number of documents published by each author, while the presence of connection lines signifies joint effort. Collaboration between authors is indicated by the number of connected lines. There are 14 distinct color clusters in total.

Cooperative relationships between authors can be more easily identified with the aid of visual mapping (Figure 5), which provides clear information about existing partnerships; authors from the top 100 researchers were used to create the visual map. The quantity of documents published by each author is represented by the diameter of the concentric circle, and the presence of connection lines denotes collaborative effort. Collaboration between authors is shown by the density of the connecting lines. In total, there are 14 clusters of different colors. In order, the largest clusters are cluster 1 (red color) and cluster 2 (green color). Cluster 1 is composed of several authors, including Bajaj, J.; Fagan, A.; Fuchs, M.; Gavis, E.; Gillevet, P.M.; Heuman, D.; Hylemon, P.B.; Sikaroodi, M.; and White, M.B. Moreover, cluster 1 shows direct cooperation with cluster 5 (blue color), which is composed of several authors including Demir, M.; Lan, S.; Loomba, R.; Martin, A.; and Scnabl, B.G. Cluster 2 is composed of several authors, including Alisi, A.; Del Chierico, F.; Gasparrini, A.; Nobili, V.; Picca, A.; Ponziani, F.R.; Putignani, L.; and Sterbini, F.P. As shown in Figure 5, some authors prefer not to constitute co-authorship and instead constitute individual clusters.

**Figure 6:**
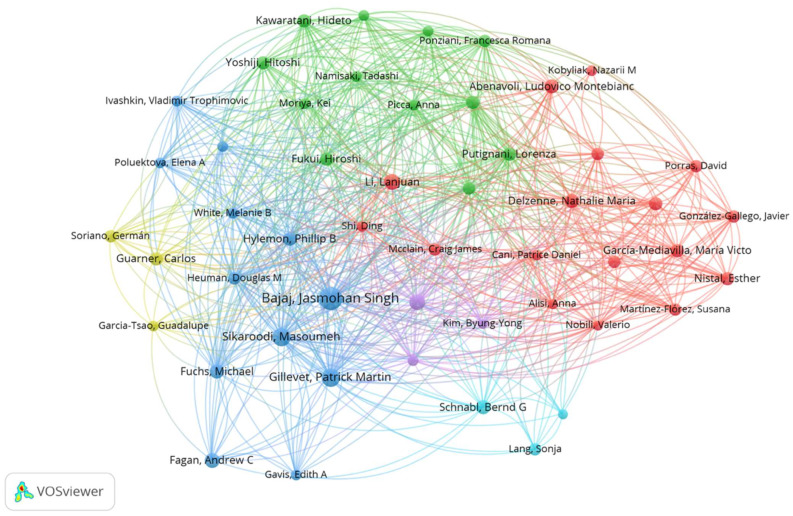
The top 50 authors’ citations were examined. The number of times authors cite each other determines their relatedness. Stronger collaborations are indicated by thicker lines. Authors represented by larger circles or larger font sizes had a higher proportion of citations. The map analysis reveals the existence of six citation clusters, each represented by a different color. Each cluster is made up of authors who cite each other the most frequently.

We also made a citation analysis of the authors, where the relatedness of the authors is determined based on the number of times, they cite each other (Figure 6). A total of 5037 authors have been cited at least once, accounting for 88.9% of the total 5663 authors; 3547 authors have had at least 10 citations (62.3%), and 749 authors have been mentioned at least 100 times (13.2%). The map analysis reveals the existence of n.6 clusters of citations represented by a different color. Each cluster is composed of authors who cite most frequently among themselves.

### 3.4. Analysis of the Most Cited Journals and Articles

Over the last 20 years, 422 journals have published a total of 1015 articles. Table 2 lists the top 20 most productive journals in terms of publications and citations related to the gut microbiome, all stages of liver disease from NAFLD to cirrhosis, and biotics. In the first three positions, the most prolific journals in order of publications (in brackets is indicated the publisher) are *Nutrients* (MDPI, Multidisciplinary Digital Publishing Institute), *International Journal of Molecular Sciences* (MDPI), and *World Journal of Gastroenterology* (Baishideng Publishing Group), while with regard to citations, in order we have *Hepatology* (John Wiley & Sons on behalf of the American Association for the Study of Liver Diseases), *Journal of Hepatology* (Elsevier), and *World Journal of Gastroenterology* (Baishideng Publishing Group). The total number of articles published in the top 20 journals was 322, constituting 31.7% of all documents retrieved. *Hepatology* has the highest number of citations among the 422 journals.

**Figure 7:**
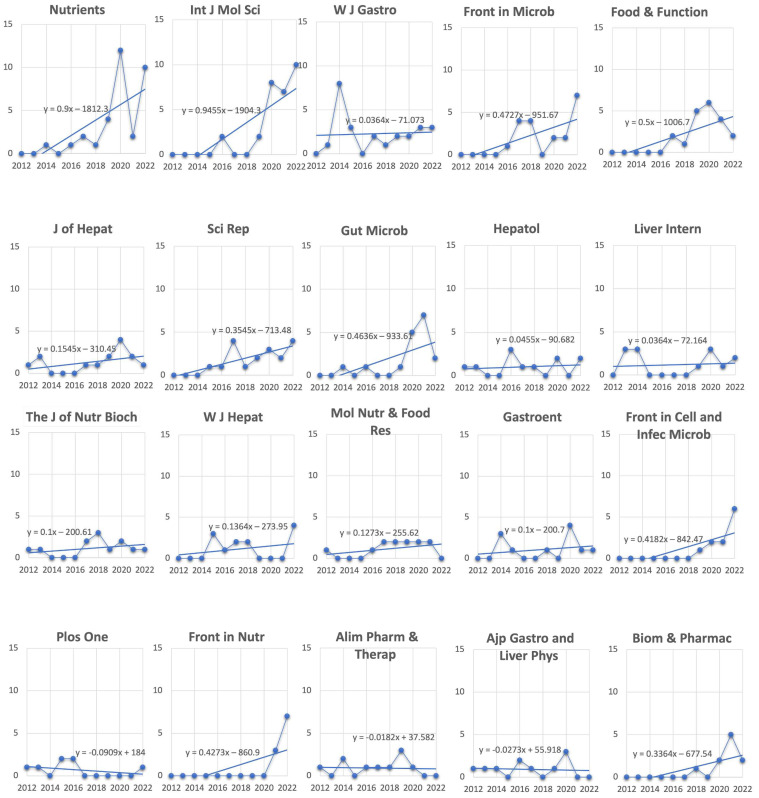
Publication trend of the 20 most prolific journals relative to the last 10 years. *Y*-axis represents the number of publications. For each graph is indicated the linear trend line and related equation. *Int J Mol Sci* = *International Journal of Molecular Sciences*; *W J Gastro* = *World Journal of Gastroenterology*; *Front in Microb* = *Frontiers in Microbiology*, *J of Hepat* = *Journal of Hepatology*; *Sci Rep* = *Science Report***;** *Gut Microb* = *Gut Microbiology*; *Hepatol* = *Hepatology*; *Liver Intern* = *Liver International*; *The J of Nutr Bioch* = *The Journal of Nutritional Biochemistry*; *W J Hepat* = *World Journal of Hepatology*; *Mol Nutr & Food Res* = *Molecular Nutrition & Food Research*; *Gastroent* = *Gastroenterology*; *Front in Cell and Inf Microb* = *Frontiers in Cellular and Infection Microbiology*; *Front in Nutr* = *Frontiers in Nutritions*; *Alim Pharm & Therap* = *Alimentary Pharmacology & Therapeutics*; *Ajp Gastro and Liver Phys* = *AJP Gastrointestinal and Liver Physiology*; *Biom & Pharmac* = *Biomedicine & Pharmacotherapy*.

As shown in Figure 7, the analysis of trend publication of each of the 20 most productive journals listed in Table 2 reveals that some journals have had a growing interest in the field in the last 10 years and among these, in particular, are *Nutrients*, *International Journal of Molecular Sciences*, *Frontiers in Microbiology*, *Frontiers in Nutrition*, and *Frontiers in Cellular and Infection Microbiology*. As for the most cited journal *Hepatology*, interest in the field remains constant over the years.

**Figure 8:**
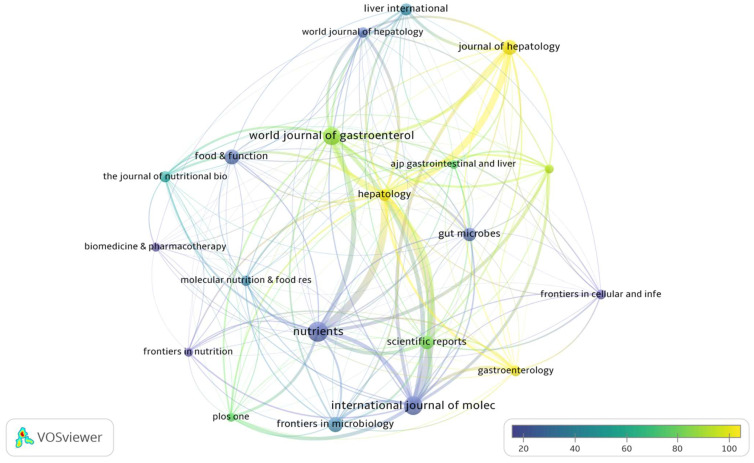
The relationship between journals is determined by the frequency with which one journal mentions another. The size of the concentric circle and the text represent the number of journal articles. The thickness of the connecting lines is proportional to the number of citations between journals. The degree of shading from dark purple to yellow varies based on the average number of citations achieved by each journal, with yellow representing the highest amount.

In Figure 8, the visual citation analysis shows the relationship in terms of citations between journals with at least 10 publications and 200 citations. The relatedness of journals is determined based on the number of times a journal cites another. The size of the concentric circle and the text denote the number of articles produced by the journals. The thickness of the connecting lines reflects the level of citation between journals. The degree of coloring from dark purple to yellow varies according to the average number of citations each journal achieved, with yellow accounting for the highest number. From the map, it can be seen that the journals that cite each other more frequently are the following: *Nutrients*, *Hepatology*, *International Journal of Molecular Sciences*, *World Journal of Gastroenterology*, *Scientific Reports*, and *Journal of Hepatology*.

The number of citations Is an important indicator of the impact of an article in a research area. The number of citations of these 1015 articles was counted and ranked, and the top 20 papers divided into reviews and original articles are provided in Table 3. Among review articles, the paper entitled “The gut microbiota and host health: a new clinical frontier” had major citations [42]. While among original articles, the study of Bounsier J. published in *Hepatology* is the most cited [43].

### 3.5. Analysis of Co-Citation References

Co-cited references are those that are cited by multiple articles from the retrieved list from the Dimensions database. Table 4 displays the top 10 citations from 30,364 references obtained from 1015 papers, ordered by citation frequency. The first-ranked reference was a clinical study published by *Hepatology* in 2013. In this study, using 16S ribosomal RNA pyrosequencing, Zou et al. investigated the composition of the gut bacterial communities of NASH patients and healthy children. They found that the increased abundance of alcohol-producing bacteria in NASH microbiomes, the elevated blood–ethanol concentration in NASH patients, and the well-established role of alcohol metabolism in oxidative stress and, consequently, liver inflammation suggest that alcohol-producing microbiota play a role in the pathogenesis of NASH. They hypothesize that the different compositions of the gut microbiota in NASH, obese individuals, and healthy controls may serve as a therapeutic target or disease marker [61]. In 2006, *Nature* published a comparative study by Turnbaugh et al., which was the second-ranked source. Changes in the relative abundance of the two dominant bacterial divisions, *Bacteroidetes* and *Firmicutes*, affect the metabolic potential of the mouse gut microbiota, according to metagenomic and biochemical analyses. Their findings indicate that the obese microbiome is better able to extract energy from food. This trait is also transmissible: colonization of germ-free mice with an “obese microbiota” results in a significantly greater increase in total body fat than colonization with a “lean microbiota” [62]. In the third-ranked reference, *Bacteroides* were found to be independently associated with NASH, and *Ruminococcus* was found to be closely associated with significant fibrosis in 57 patients with NAFLD [43]. Both the first and third articles emphasized inter-kingdom signaling, which refers to the interaction between gut bacteria and the host system, and suggested using gut microbiota as a clinical marker for diagnosing, grading, and treating NAFLD. The second article emphasized the link between gut microbiome composition and obesity which is associated with an increased risk of developing NAFLD and liver cancer progression [63,64]. The article of Mouzaki M et al., published by *Hepatology* in 2013 [54], is in the 10th position in the top 10 most-cited references and also in the top 10 cited articles (Table 4). This article is a prospective, cross-sectional study designed to discover variations in intestinal microbiota between persons with biopsy-proven NAFLD (simple steatosis or nonalcoholic steatohepatitis) and healthy liver donors as controls. The study demonstrated an inverse and BMI- and diet-independent correlation between the occurrence of NASH and the proportion of *Bacteroidetes* in the stool, suggesting that the intestinal microbiota may play a role in the development of NAFLD.

### 3.6. Dynamic Changes in Co-Occurrence Keywords: A Visualization Analysis

**Figure 9:**
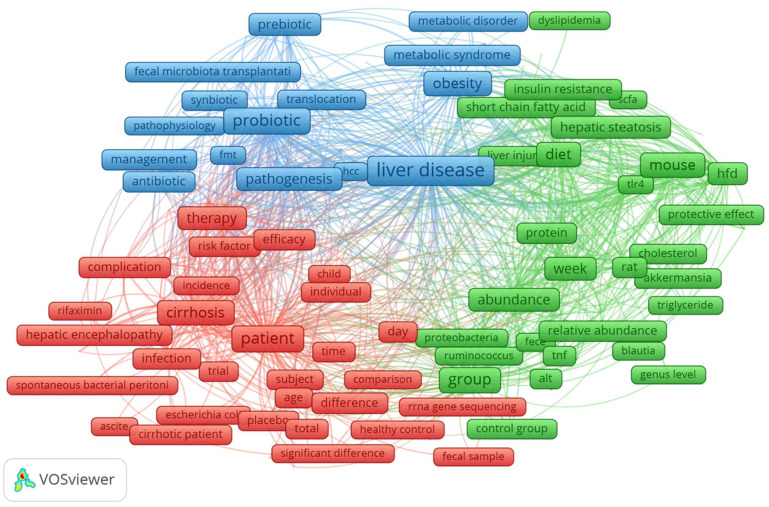
Clustered co-occurrence map of the 100 most significant terms extracted from the titles and abstracts of 1015 publications. In the analysis, only terms with 25 or more occurrences were examined. Items that are unrelated to others have been omitted. In the map, the size of the frames indicates the frequency with which the keyword occurs. Co-occurrence strength between pairs of keywords is indicated by the proximity of two nodes and the thickness of the line connecting them. The color of the frames denotes keyword clusters, which are usually composed of co-occurring terms and can be regarded as broad research subjects in the field. The greatest collection of related terms consists of 170 terms arranged into three groups, distinguishable in the three colors: red, green, and blue.

Co-occurrence analysis of keywords in the title and abstract of 1015 articles, retrieved from the Dimensions database, was conducted to reveal the most important themes and predict potential future trends. Only keywords with 25 or more occurrences were considered in the analysis. Items that are not related to others were not included. In the map, the size of the node denotes the occurrence of the keyword. The proximity of two nodes and the thickness of the line connecting them indicate the co-occurrence strength between pairs of keywords. The nodes’ color represents keyword clusters, which frequently contain co-occurring words and can be interpreted as broad research topics in the field. The size of the frames in Figure 9 represents the frequency of terms in the title and abstract. The largest collection of connected terms includes 170 terms organized into three clusters.

The cluster colored in blue includes, among the most frequent keywords, liver disease, probiotic, obesity, pathogenesis, prebiotic, synbiotic, metabolic syndrome, and disorders.

In the cluster colored in green, we found, among the most frequent keywords, group, diet, supplementation and also week, abundance, firmicute, liver inflammation, toll-like receptor-4 (TLR4), and lipid metabolism.

The cluster colored in red includes, among the most frequent keywords, cirrhosis, therapy, diagnosis, complication, and hepatic encephalopathy.

**Figure 10:**
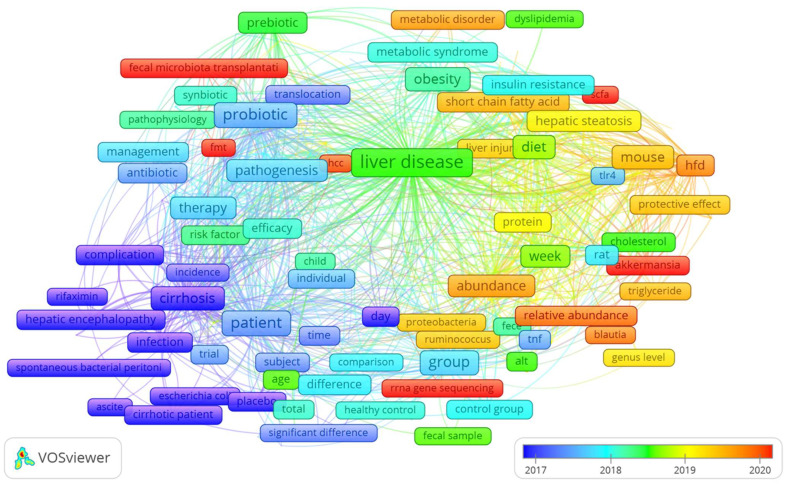
The evolution of co-occurring words over time. Overlay visualization analysis revealed a shift to the following keywords (frames in red) between 2017 and 2020: rRNA gene sequencing, *Akkermansia*, SCFA (short-chain fatty acids), lipid metabolism, and FMT (fecal microbiome transplantation).

VOSviewer analysis can identify and visualize the shift of co-occurrence words through the years. As shown in the overlay visualization in Figure 10, the analysis highlighted a shift between 2017 and 2020. We assist in a shift to the following keywords (frames in red): rRNA gene sequencing, *Akkermansia*, SCFA (short-chain fatty acids), lipid metabolism, and FMT (fecal microbiome transplantation). These results can assist researchers in speculating about the future treatment for NAFLD, its severe forms, and the progress of research on potential therapy with biotics.

## 4. Discussion

Different from previous bibliometric studies which analyze, by utilizing the Web of Sciences database, the emerging trends and hotspots in the field of metabolic-dysfunction-associated fatty liver disease (MALD) [35], the field of gut microbiota and MALD [36], and the role of the gut–liver axis in NAFLD [37], we have analyzed a selection of articles specifically concerning the relationships between the gut microbiome, all stages of liver disease, from NAFLD to cirrhosis, and their connections with biotics (prebiotics, probiotics, symbiotics, postbiotics, and probiotics). Moreover, we have examined data extracted from the free access version of the Dimensions database [38,39]. In our bibliometric analysis study, we found 1015 articles in the searched field from 2002 to 2022. The overall publication number has increased considerably in the five years. As shown in Figure 1A, we can divide the publication trend over years into three “ages”: the period from 2002 to 2009 when only 41 (4.0%) pieces of literature were published, the period from 2010 to 2016 with 231 publications (22.7%), and the flourishing period from 2017 with 741 publications (72.8%). The citation curve trend is comparable to that of publications for the period 2002–2022 (Figure 1B). China and the USA played leading roles in the field with about 49% of total publications, with China in the first place (Figure 1C). This is followed by Italy and Japan, which account for 7.3% and 6.5% of total publications, respectively. When the average number of citations per article is considered (Figure 1D), the USA holds the first place for citations, followed by China. The USA and China had a percentage of 52.8 out of a total of 43,093 citations for the period 2002–2022. Italy also holds a good number of citations (6828) representing 15.8% of the total. The USA has the best relationships with other countries in terms of cooperation (Figure 2), followed by China and Spain. The relationship between China and the USA was the closest, followed by that between the USA and Italy, Germany, and Spain. These demonstrated the United States’ important role in academic activity worldwide. There is a strong correlation between the density of producing institutions and the indicator of national output in this area, which measures the volume of publications and citations in this sector. As shown in Figure 3, four of the top ten prolific institutions are from the USA, while two are from China, also with regard to citations. Visual analysis of cooperation (Figure 4) identified a larger aggregate of 69 institutions with active collaboration, composed of nine clusters. Uncovering the underlying mechanisms will require collaboration among several organizations. Research in this area is complicated by the vast diversity of the gut microbiome and the mechanisms relating the function of the gut microbiome in the development of NAFLD into NASH and cirrhosis and its connection with biotics. The USA had 7 of the 20 most prolific authors, which explains its position among the top 10 fruitful countries. In addition, authors from Spain (4 authors), Italy (3 authors), and Japan (3 authors) are in the top 20 (Table 1). The cooperation analysis reveals that several authors of the same institution and/or country form collaboration workgroups (Figure 5). Bajaj, J. of Virginia Commonwealth University (USA) has the most works published (25), followed closely by Gillevet, P.M. and Sikaroodi, M. of George Mason University (USA) (each with 15). In third place, with 12 publications each, are researchers Su, K.T. of Hallym University (Republic of Korea) and Li, L. of Zhejiang University (China). When looking at the top 20 cited writers, the ones with the most citations are, once again, Bajaj, J. (2375), Gillevet, P.M. (1932), Sikaroodi, M. (1932), Suk, K.T. (1347), and Hylemon, P.B. (USA, 1347). These authors published the most on the relationship between the gut microbiome and NAFLD and its advanced pathological forms, as well as biotics. Although, as stated above, both the source of the data and the research topics are different from some major previous bibliometric papers, data on publication trends and productivity/cooperation of nations are comparable to the results of these previous publications [35,36,37]. The journal with the most publications was *Nutrients* (33), followed by the *International Journal of Molecular Sciences* (29), the *World Journal of Gastroenterology* (27), *Frontiers in Microbiology* (20), *Food & Function* (20), and the *Journal of Hepatology* (20). The two most prolific journals, *Nutrients* and *International Journal of Molecular Sciences*, publish articles on a broad range of topics, such as diet-related disorders, metabolic syndrome, nutritional supplements, molecular microbiology, molecular metabolism, molecular pathology, diagnostics and therapeutics, bioactives, and nutraceuticals. *Hepatology* had the greatest number of citations (4555), followed by the *Journal of Hepatology* (2348), the *World Journal of Gastroenterology* (2279), *Gut* (2002), *Scientific Reports* (1439), and *Gastroenterology* (1179). *Hepatology* and the *Journal of Hepatology*, two of the most cited journals, publish articles on all areas of liver structure, function, and illness. Of the above-mentioned journals, only a few have exhibited a notable growth pattern during the past 10 years (Figure 7). *Nutrients*, *International Journal of Molecular Sciences*, *Hepatology*, *Journal of Hepatology*, and also *World Journal of Gastroenterology* and *Scientific Reports* are the journals that cite each other more frequently, demonstrating their prominence in the field (Figure 8). Dysbiosis of the gut microbiome plays a significant role in food supply and balance [1,71], but it also contributes to the pathogenic processes of several diseases [72,73,74]. There is mounting evidence from both preclinical and clinical research indicating that disturbance of the gut–liver axis plays a central role in NAFLD etiology and its advanced stages, and that gut dysbiosis is a key factor in the development of this dysfunction [12,13]. Therefore, research on the role of the gut microbiome in the course of NAFLD disease and the use of biotics to reduce dysbiosis fits well within the scope of the journals listed above. The first most cited review article was authored by Marchesi and published by the journal *Gut* in 2015 [42] (Table 3). The paper focused on the potential of manipulating the gut microbiota in metabolic syndrome and obesity-related disease, liver disease, inflammatory bowel disease, and colorectal cancer, with an examination of the most recent and relevant evidence relating to antibiotics, probiotics, prebiotics, polyphenols, and fecal microbiota transplantation. The article has inspired research in the field, which has been an exponential increase since 2016. Instead the study by Bounsier et al. [43] (Table 3) published in *Hepatology* is the most-cited original paper. The authors examined the relationship between gut dysbiosis and severity of NAFLD lesions in a well-defined adult NAFLD cohort. They discovered that the severity of NAFLD is connected with gut dysbiosis and a change in the metabolic function of the gut microbiota. In addition, they identified *Bacteroides* as being independently associated with NASH and *Ruminococcus* with severe fibrosis.

Table 4 lists the top 10 references from 30,364 retrieved from 1015 publications. The top-ranked reference was a clinical trial published in *Hepatology* by Zhu et al. [61]. In this article, thorough epidemiological information on NASH is offered to highlight its significance. The author proposed that the unique composition of the gut microbiome between NASH and healthy controls could serve as an intervention target or a disease marker. Table 4′s highest cited references may be the most widely accepted studies in the field because of the significant contributions they made in elucidating the link between gut microbiome in the progression of NAFLD, into NASH and cirrhosis, and its connection with biotics.

Beyond the data relative to productivity cooperation between countries, institutes, and authors or the relevance of journals and most cited studies, one of the most interesting aspects of a bibliometric analysis is related to emerging topics. Using co-occurrence word analysis, we can visualize the most important themes and predict potential future trends.

The terms *obesity* and *metabolic syndrome*/*disorders* (Figure 9) appear among frequent keywords in clusters colored in blue. There is evidence linking dysbiosis in the gut microbiome to metabolic illnesses such as obesity and chronic liver diseases [72,73]. The risk of developing NAFLD increases in tandem with the body mass index [63,75,76]. Dysbiosis due to obesity contributes to the development of NAFLD [77]. The prevalence of obesity continues to rise, making it a serious threat to public health in Western cultures [78,79]. Sedentary lifestyles and excessive food intake generate a nutritional imbalance, which contributes to the prevalence of obesity, especially among those who are genetically susceptible to the condition [80,81]. These emerged topics are according to the bibliometric studies of Liao and Li which reveal interesting hotspots of metabolic-associated fatty liver disease, obesity, and children/adolescent who suffer from obesity-related illnesses at an increasing rate [35,36].

As shown in Figure 9, *probiotic*, *prebiotic*, and *synbiotic* are also frequent keywords. Several pieces of evidence have demonstrated that probiotics and prebiotics can reduce dysbiosis [42,53,82,83,84,85]. The probiotic *Lactobacillus* has been highlighted as a research hotspot [36]. Synbiotics, which have positive effects on the balance of gut microbiota, are a combination of prebiotics and probiotics [14,19,86]. Synbiotics have been shown to ameliorate NAFLD severity [19,86]. Inflammation, obesity, and changes in glucose and lipid metabolism are some of the main causes of NAFLD; however, the exact pathophysiological processes and mechanisms that lead to NAFLD are not well understood. There is some evidence that synbiotics can help people with NAFLD reduce inflammation and improve insulin resistance [16,84,87]. The keywords *postbiotics* and *parabiotics* did not appear among frequent terms, probably because these topics are still relatively new.

In the cluster colored in green, we found *group*, *diet*, *supplementation*, *week*, *abundance*, *firmicute*, *liver inflammation*, and *toll-like receptor-4* (*TLR4*). The diversity, composition, and metabolic activity of the gut microbiome are highly associated with dietary patterns and nutrient intake [88,88]. Dysbiosis in patients with NAFLD, NASH, and cirrhosis is characterized by a decrease in total bacterial diversity and richness as well as a shift toward a community composed of more *Firmicutes* and fewer *Bacteroidetes* [89,90,91]. Moreover, dietary modifications alter the composition and activity of the gut microbiome, which may assist in ameliorating comorbidities, such as metabolic syndrome and NAFLD, and delaying their progression into more severe forms, such as NASH and cirrhosis [92,93,94]. As aforementioned, inflammation is one of the main causes of NAFLD, and the severity of inflammation influences its progression into more severe forms [95]. Toll-like receptors (TLRs) are activated by components of gut bacteria, leading to liver inflammation and ultimately the development of NASH and NASH-related cancer [96,97,98]. The expression of TLR ligands in the gut is reduced by the use of probiotics [99,100,101]. Similarly, the bibliometric analysis of Yang reveals that the inflammation theme is an active research topic [37].

The terms *cirrhosis*, *therapy*, *diagnosis*, *complication*, and *hepatic encephalopathy* appear among the frequent keywords in the cluster colored blue. The diagnosis and treatment of cirrhosis, the last stage of alcohol-related liver disease, are crucial [94,102]. On the other hand, the search for noninvasive markers and avoiding the development of liver cancers are emerging hotspots of fundamental importance [35]. Hepatic encephalopathy is a common consequence of cirrhosis that causes cognitive and movement impairment. The pathogenesis of hepatic encephalopathy is significantly influenced by microbiome–host interactions [103,104].

As shown in Figure 10, from the year 2017 to 2020, we assisted in a shift to the keywords (frames in red): rRNA gene sequencing, *Akkermansia*, *SCFA* (*short-chain fatty acids*), and *FMT* (*fecal microbiome transplantation*).

The rRNA sequencing is an important tool for identifying the bacterial communities that constitute the microbiome, and its use has expanded from the laboratory to the clinic in recent years [105,106]. Under pathological conditions such as NAFLD, and as it progresses to more severe forms such as NASH and cirrhosis, we can analyze the abundance and classification of bacteria by sequencing the DNA sequences that encode ribosomal 16S rRNA in the bacterial genome and learn about meaningful changes of the microbiome [107]. It can also be used to create new probiotics and study the effects of biotics on the gut microbiome.

Recently, *Akkermansia muciniphila*, a novel microbe that regulates numerous metabolites, including SCFA, has been added to the list of probiotics based on results from a variety of animal and human investigations [108,109]. Treatment with *Akkermansia muciniphila* decreased insulin sensitivity, fat deposition, and weight gain in animal models [110]. Supplementation with *A. muciniphila* lowers hepatic steatosis and intestinal permeability [111]. Amuc 1100, a protein derived from *A. muciniphila*’s outer membrane, has been shown to interact with TLRs [112].

Dietary fiber and indigestible starch are broken down by gut microbiota into SCFAs, the types of which vary with the kind of microbiota present and the type of fiber eaten [113]. More than 90% of the SCFAs in the digestive tract are composed of acetate, propionate, and butyrate [114]. The liver uses SCFAs as an energy source and as a key player in lipogenesis and gluconeogenesis after they are absorbed in the intestine [115]. Reduced levels of butyrate and butyrate-producing bacteria are frequently related to metabolic problems [116], indicating that the precise distribution of SCFAs may be crucial for understanding their role in NAFLD. Elevated amounts of fecal acetate were found in individuals with severe fibrosis, while increased levels of fecal butyrate and propionate were seen in patients with mild to moderate fibrosis [117,118]. The importance of SFCAs as an object of future research is an emerging hotspot [37].

Reconstructing and restoring the intestinal microecological balance and diversity using FMT is an innovative technique. FMT has been shown to be effective in the treatment of a variety of conditions, including hepatic encephalopathy [103,119,120,121]. Several studies in animals have indicated that FMT can ameliorate NAFLD, NASH, and cirrhosis symptoms by rebalancing the gut microbiome [26,122,123]. Due to its promising results, FMT is increasingly being considered for use in NAFLD/NASH and cirrhotic patients. The interaction between gut microbiota composition and NAFLD was also highlighted by the findings of Witjes et al. [124], who found that FMT from healthy donors could influence hepatic gene expression as well as plasma metabolites implicated in inflammation and lipid metabolism. Therefore, the development of microbiome-targeted therapeutic techniques should be investigated to facilitate the discovery of novel preventive and therapeutic approaches for NAFLD and derived pathological spectrum.

While every effort was made to be as thorough as possible and make sure the results were accurate, there were still some limitations. First, we examined only articles in the free access version of the Dimensions database. It is possible that if we had used a different set of databases, we might have gotten different results. Secondly, we limited our search to articles originally published only in the English language, overlooking some studies published in other languages.

## 5. Conclusions

The role of the gut microbiome in the progression of nonalcoholic fatty liver disease (NAFLD) and its relationship with biotics has become a hot topic in recent years. Over the past decade, there has been a significant increase in studies in this field. China leads the field with the biggest number of papers, followed closely by the United States. However, the United States is the most influential country in this area. As such, it is essential for authors and nations to work closely together in order to advance the area. Out of a total of 422 journals, most articles have been published in *Nutrients*. *Hepatology* had the greatest number of citations. Research into the following topics is expected to emerge in this field:

- evaluation of risk factors that are associated with the progression of NAFLD, such as obesity and metabolic syndrome;
- pathogenic mechanisms, such as liver inflammation through toll-like receptor activation or alteration of short-chain fatty acid metabolisms, which contribute to NAFLD development and its progression in more severe forms, such as cirrhosis;
- therapy for cirrhosis rough dysbiosis reduction, and research on hepatic encephalopathy, a common consequence of cirrhosis;
- evaluation of diversity, and composition of gut microbiome under NAFLD, and as it varies under NASH and cirrhosis by rRNA gene sequencing, a tool which can also be used for the development of new probiotics and explore into the impact of biotics on the gut microbiome;
- treatments to reduce dysbiosis, almost with probiotics, prebiotics, and synbiotics, and with new probiotics, such as *Akkermansia*, or FMT, or diet.

These findings have significant implications for future research into the role of the gut microbiome in the progression of NAFLD and its connection with biotics.

## Figures and Tables

**Table 1:** The top 20 authors in order of publication numbers. All numbers are derived from Dimensions on 25 January 2023.

Name	Organization, Country	Publications	Citations	Citations (Mean)
Jasmohan Singh Bajaj	Virginia Commonwealth University, United States	25	2375	95
Patrick Martin Gillevet	George Mason University, United States	15	1932	128.8
Masoumeh Sikaroodi	George Mason University, United States	15	1932	128.8
Ki Tae Suk	Hallym University, Republic of Korea	12	260	21.67
Lanjuan Li	Zhejiang University, China	12	635	52.92
Andrew C. Fagan	Virginia Commonwealth University, United States	11	153	13.91
Nathalie Maria Delzenne	Université Catholique de Louvain, Belgium	11	702	63.82
Bernd G. Schnabl	University of California, San Diego, United States	10	385	38.5
Michael Fuchs	Virginia Commonwealth University, United States	10	877	87.7
Ludovico Montebianco Abenavoli	Magna Graecia University, Italy	9	332	36.89
Phillip B. Hylemon	Virginia Commonwealth University, United States	8	1347	168.38
María Victoria García-Mediavilla	Centro de Investigación Biomédica en Red de Enfermedades Hepáticas y Digestivas, Spain	8	462	57.75
Carlos Guarner	Hospital de Sant Pau, Spain	8	711	88.88
Hitoshi Yoshiji	Nara Medical University, Japan	8	290	36.25
Esther Nistal	University of Leon, Spain	8	462	57.75
Hideto Kawaratani	Nara Medical University, Japan	8	291	36.38
Antonio Gasbarrini	Catholic University of the Sacred Heart, Italy	8	534	66.75
Lorenza Putignani	Bambino Gesù Children’s Hospital, Italy	8	896	112
Hiroshi Fukui	Nara Medical University, Japan	8	513	64.13
Sonia Sánchez-Campos	Centro de Investigación Biomédica en Red de Enfermedades Hepáticas y Digestivas, Spain	8	462	57.75

**Table 2:** List of the 20 most productive journals in terms of publications and citations, in descending order. All numbers are derived from Dimensions on 25 January 2023.

Journal	Documents	Journal	Citations
*Nutrients*	33	*Hepatology*	4552
*International Journal of Molecular Sciences*	29	*Journal of Hepatology*	2348
*World Journal of Gastroenterology*	27	*World Journal of Gastroenterology*	2279
*Frontiers in Microbiology*	20	*Gut*	2002
*Food & Function*	20	*Scientific Reports*	1437
*Journal of Hepatology*	20	*Gastroenterology*	1178
*Scientific Reports*	18	*Alimentary Pharmacology & Therapeutics*	894
*Gut Microbes*	17	*Nutrients*	799
*Hepatology*	15	*AJP Gastrointestinal and Liver Physiology*	756
*Liver International*	15	*PLoS ONE*	755
*The Journal of Nutritional Biochemistry*	13	*Frontiers in Microbiology*	728
*World Journal of Hepatology*	12	*International Journal of Molecular Sciences*	723
*Molecular Nutrition & Food Research*	12	*The Journal of Nutritional Biochemistry*	683
*Gastroenterology*	12	*Clinical Gastroenterology and Hepatology*	667
*Frontiers in Cellular and Infection Microbiology*	11	*Liver International*	599
*PLoS ONE*	10	*Journal of Clinical Gastroenterology*	598
*Frontiers in Nutrition*	10	*Food & Function*	530
*Alimentary Pharmacology & Therapeutics*	10	*Molecular Nutrition & Food Research*	454
*AJP Gastrointestinal and Liver Physiology*	10	*Digestive Diseases and Sciences*	450
*Biomedicine & Pharmacotherapy*	10	*Gut Microbes*	439

**Table 3:** The 20 most-cited publications, sorted in descending order by review articles and original articles. All numbers are derived from Dimensions on 25 January 2023.

**Reviews**					
**Title**	**Source Title**	**PubYear**	**Authors**	**Times Cited**	**Ref**
The gut microbiota and host health: a new clinical frontier	*Gut*	2015	Marchesi, Julian R	1436	[42]
Bacterial translocation (BT) in cirrhosis	*Hepatology*	2005	Wiest, Reiner	569	[44]
The role of the gut microbiota in NAFLD	*Nature Reviews Gastroenterology & Hepatology*	2016	Leung, Christopher	561	[45]
The gut-liver axis in liver disease: pathophysiological basis for therapy	*Journal of Hepatology*	2019	Albillos, Agustín	546	[12]
Mechanisms of action of probiotics	*Advances in Nutrition*	2019	Plaza-Diaz, Julio	446	[46]
Diabetes, obesity and gut microbiota	*Best Practice & Research Clinical Gastroenterology*	2013	Everard, Amandine	421	[47]
The role of the gut microbiota in nonalcoholic fatty liver disease	*Nature Reviews Gastroenterology & Hepatology*	2010	Abu-Shanab, Ahmed	377	[48]
Gut microbiota and metabolic syndrome	*World Journal of Gastroenterology*,	2014	Festi, Davide	349	[49]
Gut microbiome and liver diseases	*Gut*	2016	Tilg, Herbert	322	[50]
Alcohol, liver disease and the gut microbiota	*Nature Reviews Gastroenterology & Hepatology*	2019	Bajaj, Jasmohan S	299	[51]
**Original articles**					
**Title**	**Source Title**	**PubYear**	**Authors**	**Times Cited**	**Ref**
The severity of nonalcoholic fatty liver disease is associated with gut dysbiosis and shift in the metabolic function of the gut microbiota	*Hepatology*	2016	Boursier, Jérôme	807	[43]
The gut microbiome in atherosclerotic cardiovascular disease	*Nature Communications*	2017	Jie, Zhuye	784	[52]
Probiotics and antibodies to TNF inhibit inflammatory activity and improve nonalcoholic fatty liver disease	*Hepatology*	2003	Li, Zhiping	747	[53]
Intestinal microbiota in patients with nonalcoholic fatty liver disease	*Hepatology*	2013	Mouzaki, Marialena	540	[54]
Modulation of the fecal bile acid profile by gut microbiota in cirrhosis	*Journal of Hepatology*	2013	Kakiyama, Genta Ridlon	530	[55]
Fecal microbiome and volatile organic compound metabolome in obese humans with nonalcoholic fatty liver disease	*Clinical Gastroenterology and Hepatology*	2013	Raman, Maitreyi	495	[56]
Synbiotic modulation of gut flora: Effect on minimal hepatic encephalopathy in patients with cirrhosis	*Hepatology*	2004	Liu, Qing	493	[57]
Gut microbiota profiling of pediatric nonalcoholic fatty liver disease and obese patients unveiled by an integrated meta-omics-based approach	*Hepatology*	2016	Del Chierico, Federica	424	[58]
Colonic mucosal microbiome differs from stool microbiome in cirrhosis and hepatic encephalopathy and is linked to cognition and inflammation	*AJP Gastrointestinal and Liver Physiology*	2012	Bajaj, Jasmohan S	397	[59]
Dysbiosis gut microbiota associated with inflammation and impaired mucosal immune function in intestine of humans with non-alcoholic fatty liver disease	*Scientific Reports*	2015	Jiang, Weiwei	387	[60]

**Table 4:** The 10 most cited references, in descending order. All numbers are derived from Dimensions on 25 January 2023.

Title	First Author	Citations	Journal	Year	Ref
Characterization of gut microbiomes in nonalcoholic steatohepatitis (NASH) patients: a connection between endogenous alcohol and NASH	Zou, L.	207	*Hepatology*	2013	[61]
An obesity-associated gut microbiome with increased capacity for energy harvest	Turnbaugh, P.J.	143	*Nature*	2006	[62]
The severity of nonalcoholic fatty liver disease is associated with gut dysbiosis and shift in the metabolic function of the gut microbiota	Boursier, J.	135	*Hepatology*	2016	[43]
Increased intestinal permeability and tight junction alterations in nonalcoholic fatty liver disease	Miele, L.	129	*Hepatology*	2009	[65]
Altered profile of human gut microbiome is associated with cirrhosis and its complications	Bajaj, J.S.	115	*Journal of Hepatology*	2013	[66]
The gut microbiota as an environmental factor that regulates fat storage	Bäckhed, F.	113	*PNAS USA*	2004	[67]
Characterization of fecal microbial communities in patients with liver cirrhosis	Chen, Y.	110	*Hepatology*	2011	[68]
Metabolic endotoxemia initiates obesity and insulin resistance	Cani, P.D.	109	*Diabetes*	2007	[69]
Alterations of the human gut microbiome in liver cirrhosis	Qin, N.	108	*Nature*	2014	[70]
Intestinal microbiota in patients with nonalcoholic fatty liver disease	Mouzaki, M.	100	*Hepatology*	2013	[54]

## Data Availability

Raw and processed data are available upon request to the corresponding author.
